# How to Live Long

**Published:** 1889-01

**Authors:** 


### HOW TO LIVE LONG.

The true secret of good health and immunity from disease lies in finding out and practising the golden mean of every creed. The vegetarian, for instance, goes too far ; but he is perfectly correct in his assumption that most men eat too frequently and too plentifully of meat, and not nearly enough of vegetables and fruit. The average Englishman believes in good slices from the joint, usually underdone, and often eaten in haste, with the day's work but half done. Vegetables are with him a very secondary consideration, partly because they are often badly cooked and not temptingly served. Were he to eat less meat and more vegetables and fruit he would be less of a martyr to rheumatism in his old age than he is at present. Nor is he sufficiently appreciative of fish as an article of diet. Here, again, unsatisfactory cooking comes in as a factor in deterring the general public from what is good for them. The ordinary English cook is as wasteful in her methods of cooking it as she is careless in her manner of serving it. The man who does the most justice to his own constitution is he who compasses an attractive variety in his diet, ranging through all the flavors of fish, flesh, fowl, and the wares of the greengrocer in a way that not only satisfies appetite, but stimulates it.

Tea or coffee taken with meat is simply suicidal. These hot beverages turn the meat into something resembling leather, and the result interferes sadly with digestion. The man who desires long life must not give a place to "high tea" in his daily programme. Of tea itself it can only be said that it is harmless if not taken too often or made too strong. The American lady who after several calls and a cup of tea at each remarked that she could "always worry down another cup," was probably unaware of the mischief she was doing herself. No one need totally abstain from tea if they will take the precaution to buy it good, not to make it strong, not to let it infuse long, never to take it more than twice a day, and to abjure it after five in the afternoon.

As to the man for whose bath the ice has to be broken on the Serpentine on winter mornings, who can deny that he is intemperate in the matter of cold water? And yet the morning tub is indispensable to all who wish to live a long and healthy life. It is true that there have been centenarians who have known nothing of this luxury, but their longevity has been in spite of that fact, not because of it. The bath is good, but not too much bath. Walking is good, but it must not be overdone. Dickens overdid it. Most of us, however, underdo it, and scarcely walk enough. Flesh accumulates upon us in middle age because we do not take sufficient exercise, and then we give up long walks because we are stout and consequently lazy, thus reversing the process of cause and effect. The health suffers seriously, and the way is opened to many maladies. People who assert that they have not time to take long walks should remember that they are probably cutting short their own time by refraining from the needful exercise. Many people take too much medicine. Morbid persons with hypochondriacal tendencies are always dosing themselves. They apparently regard their own interior arrangement as a sort of puzzle that has been badly put together, and their efforts to sort things out with the aid of pills and powders are but a series of experiments. Highly destructive to cheerfulness is this frame of mind, and cheerfulness is one of the best ends to length of days. It is possible to cultivate this quality and in the interests of those about us, no less than in our own, it ought to be cultivated. It is a sign of a healthy mind, and enables its possessor in a certain degree to shake off worry, which is a terrible shortener of human life. No one ever died of work, but worry has killed its thousands. There are many ways of avoiding it. The chief is to live within one's income, and thus escape the wearing cares that come of debt and improvidence, avoiding anxiety for the future of those dependant on us. A little voluntary self-denial saves a mountain of it, enforced and inevitable, just as the proverbial "stitch in time saves nine." —*London Daily News.*

